# In search of universal health coverage: the hidden cost of family planning to women in Ghana

**DOI:** 10.1186/s13104-020-4928-2

**Published:** 2020-02-07

**Authors:** John Amissah, Emmanuel Kwaku Nakua, Eric Badu, Alexander Baba Amissah, Leticia Lariba

**Affiliations:** 1 Department of Health Policy Planning, Management and Economics, School of Public Health, Kwame Nkrumah University of Science and Technology, Kumasi, Ghana; 2 Department of Epidemiology and Biostatistics, School of Public Health, Kwame Nkrumah University of Science and Technology, Kumasi, Ghana; 3 Department of Nursing, University of Newcastle, Callaghan, Australia; 4 Institute of Continuous Education and Distance Learning, University of Ghana, Legon, Accra, Ghana; 5 Department of Pharmaceutics, College of Health Sciences, Kwame Nkrumah University of Science and Technology, Kumasi, Ghana

**Keywords:** Family planning, Contraceptives, Direct cost, Indirect cost, Ghana

## Abstract

**Objective:**

This study aims to estimate the cost of accessing FP services in Ghana. A cross-sectional design, involving quantitative methods were used to recruit 1194 women who accessed FP services in 336 primary health facilities, selected through a two-stage sampling. Descriptive statistics were used to estimate the cost, using STATA 13.

**Results:**

The average age of the women was 29 ± 6.87. Most women had basic education. The sources of payment for FP services were self-finance, family and sponsorship. The average direct cost of accessing FP services was GHS 7.90 [US$ 1.76]. The cost of FP services was highest for consultation GHS 7.50 [US$ 1.67], Laboratory test/x-ray GHS 6.03 [US$ 1.34], Transportation GHS GHS5.50 [US$ 1.22], Contraceptive GHS 4.73 [US$ 1.05] and Client records Card GHS 3.30 [US$ 0.73]. The cost of FP services was higher for clients visiting private facilities, tertiary level as well as those in urban centers. Clients on average spent 54.21 min traveling at a distance of 3.49 km and wait averagely 18.11 min for each visit. Government stakeholders are encouraged to revise the existing maternal health policies, as well as increase the list of FP services within the exemption package of the NHIS policy.

## Introduction

Improving family planning (FP) services and contraceptive uptake coverage in low-middle-income countries are topical public health concerns. Estimates suggest that access to modern contraceptives in developing countries could reduce the problem of unmet need for contraceptive uptake and reproductive health services, avert over 54 million unwanted pregnancies and 26 million abortions worldwide each year [1, 2]. However, the projection on achieving 65% global coverage is yet to be realized [1]. With the current growth trends, the unmet needs for FP are expected to rise from 900 million women in 2010 to 962 million women by 2020 [3].

Past studies have shown that FP reduces the need for unsafe abortion [1, 2]. Family planning reinforces people’s rights to determine the number and spacing of their children. Attempts to achieve an increment in the number of FP users have been plagued with a number of access barriers, such as accessibility (e.g. proximity), availability (e.g. commodity stockout), awareness (e.g. socio-cultural issues, education and health literacy) and affordability of services [4–12]. These barriers progressively slow efforts to improve FP uptake and coverage [3, 13]. The health systems in most developing economies rely on out of pocket payment for certain health services. Here, consumers sometimes bear as high as 80% of the total medical cost [14, 15]. This high cost of care means that consumers may turn to other cost-saving and non-conventional way of care, which may later result in some undesirable outcomes [16–18]. This makes cost a major barrier to health-seeking behavior among consumers of FP services [17, 19–22].

In Ghana, contraceptive uptake has been projected to reach at least 50% coverage by the year 2020 [23]. The current contraceptive rate remains around 22–26% among married women [24]. The government of Ghana has made several attempts to improve access to FP services. For instance, the National Health Insurance Scheme (NHIS) policy was introduced in 2005 to remove financial barriers to health services including FP. In addition, free maternal health services were introduced in 2008 to increase access to maternal health services for pregnant women [20, 25, 26]. Notwithstanding that, a large number of women are unable to access FP services due to some cost burden. Several studies have recently been undertaken regarding universal health financing for health services, including FP. These studies have addressed largely general health services and maternal health services [26–30]. Despite this, little is known about the cost burden of accessing FP services among women. The few studies on cost burden target other public health issues such as occupational injuries [31, 32] and malaria [33, 34]. To our knowledge, no study has estimated the cost burden of family planning services in Ghana. This study, therefore, aims to contribute to the literature by estimating the direct and indirect cost of accessing FP services in Ghana.

## Main text

### Methodology

A facility-based cross-sectional survey was conducted among women seeking family planning services across selected health facilities in the administrative regions of Ghana from December 2017 to January 2018. The country has an estimated population of 30 million and operates a 3-tier health system, being the tertiary, secondary and primary levels [35, 36]. Tertiary levels are the teaching hospitals whereas the regional and district hospitals belong to the secondary level, whiles all other service delivery points (SDPs) below the above mentioned are classified as primary level. Multi-Stage stratified sampling was used to recruit 1194 women seeking FP services.

The first stage involved stratification of each of the three health facility levels in the ten (10) administrative regions of Ghana, creating a total of 30 sampling stratum. Implicit stratification with proportional allocation was achieved at each level by using a probability proportional to size selection procedure (Additional file 1: Table S1).

A sample of 336 SDPs was independently selected. Sampling weights were computed for the analysis to ensure the actual representation of the population at both national and district levels. At the SDPs, exiting FP clients were enrolled and those who consented were interviewed.

#### Data collection

Data on the socio-demographics (e.g. age, sex, marital status, educational level and place of residence) and the cost incurred by clients (e.g. transportation cost, cost of FP method, and waiting time) seeking FP services were collected using a structured questionnaire by trained resident enumerators (RE). The questionnaire was programmed and loaded onto a smartphone with ODK (Open Data kit) [37, 38]. A face-to-face interview was conducted in the respondent’s preferred local dialects (English, Twi, Ga, Ewe, Dagbani, and Frafra). REs were trained on the use of ODK application for data collection and translation of the questions into their respective languages. The questionnaire was pre-tested at Ejisu-Jauben Municipality in a facility that was not sampled for the actual study.

#### Data analysis

A prevalence-based cost of illness approach focusing on direct cost and lost time value was employed to estimate the cost of seeking family planning services. This method has widely been adopted in estimating the cost of under-reported public health issues in several studies [31, 39]. Data were analyzed using Microsoft Excel and STATA version 14. Data were summarized into means, frequencies, median and percentiles. The direct cost was estimated using approaches adopted in earlier studies on birth-related mortalities and occupational injuries [31, 40–42]. It was estimated by summing all cost incurred by clients in seeking FP service at the SDP:

- Direct cost = folder fee + consultation fee + laboratory/X-ray charges + commodity purchased + transportation cost.
- Indirect Cost (Time value loss) = summation of traveling time, distance traveled and waiting time at SDP for FP services.

The time value lost was estimated on the assumption that every hour lost owing to seeking FP services contributes to productivity loss.

All monetary estimates were expressed in Ghanaian Cedis and US dollars at an exchange rate of GHS 4.50 = US$ 1 as published by the central bank in December 2017.

### Results

The mean age of the clients was 29.1 ± 6.9, more than half (53.3%) were in the age range of 25–34 years. Almost all clients were female (99.6%) and about 58.0% were currently married, while 3.94% were divorced. About 53.4% of the clients had up to junior high school level, 3-in-4 were urban residents and 47.7% access the SDPs by walking. Fifty-six percent of clients were required to visit the SDPs once every 3 months, while a few others had to visit every 5 years (6.8%). The majority of the clients (88.4%) paid for the methods received and this payment was made by themselves (56.8%), spouse (68.8%) and other family members (1.3%) (Table 1).

**Table 1:** Socio-demographic characteristics

Characteristics	*n *= 1194	%
Age group (years)		
14–24	307	25.7
25–34	639	53.3
35–44	213	17.8
45–49	30	2.5
> 50	5	0.4
Mean (SD)	29.10 (6.87)	
Sex		
Female	1189	99.6
Male	5	0.4
Marital status		
Currently married	693	58.0
Co-habitation	286	24.0
Single	168	14.1
Divorced	47	3.9
Educational level		
No formal education	209	17.5
Basic education*	638	53.4
High school	174	14.6
Tertiary**	173	14.5
Nature of residence		
Rural	317	26.5
Urban	878	73.5
Means of transport		
Walking	570	47.7
Bicycle/motorbike	162	13.6
Bus/taxi	426	35.7
Personal/private vehicle	36	3.0
Frequency of SDP visit for Family planning services		
Once a month	264	22.6
Once every 3 months	662	56.6
Once every 3 years	165	14.1
Once every 5 years	79	6.8
Paid for FP method		
Yes	1055	88.4
No	139	11.6
Financing FP method		
Self		
Yes	678	56.8
No	516	43.2
Spouse		
Yes	373	68.8
No	821	31.2
Family		
Yes	16	1.3
No	1178	98.7

* Basic education: Nine-year training from primary one to completion of junior high school and middle school

** Tertiary education: includes university, polytechnic and training colleges

#### Cost burden for accessing family planning services

The study disaggregated the burden of accessing FP by the difference in residential patterns, SDP ownership, level of SDP visited and the means of transportation used (Table 2). Clients who visited private facilities incurred the highest average cost of GHS 10.90 [$2.42], while those visiting faith-based facilities incurred the least cost of GHS 4.30 [$0.96]. Accessing care in Tertiary level facilities was high in cost compared to primary level facilities (average GHS 11.10 [$2.47] vs GHS 6.60 [$1.47]). On average, the cost was higher in urban centers (GHS 7.50 [$1.67]) compared with rural areas (GHS 6.80 [$1.51]). The average cost was high for clients who paid for the FP methods (GHS 7.90 [$1.76]); users of private vehicles as means of transportation to SDPs (GHS 23.00 [$5.11]) and for methods which were paid for by family members other than spouses (GHS10.09 [$2.24]).

**Table 2:** Cost burden: Cost by SDP ownership and type, residential pattern and means of transportation used in accessing FP services

Variables	*n (*%)	Total cost GHS ± SD	Total cost USD ± SD	Average GHS	Average USD	Median GHS	Median USD	IQR GHS	IQR USD
Service delivery point (SDP) ownership									
Government public facility	944 (80.61)	6,661.30 ± 9.2	1,480.29 ± 2.04	7.10	1.58	5.00	1.11	7.00	1.56
Private facility	141 (12.04)	1,535.90 ± 3.6	341.31 ± 0.80	10.90	2.42	5.00	1.11	5.00	1.11
Faith-based facility	86 (7.34)	370.00 ± 4.5	82.22 ± 1.00	4.30	0.96	2.00	0.44	3.50	0.78
SDP level									
Primary	655 (55.94)	4,293 ± 14.4	954.00 ± 3.20	6.60	1.47	4.00	0.89	6.00	1.33
Secondary	459 (39.20	3,642.1 ± 16.2	809.36 ± 3.60	8.00	1.78	5.00	1.11	7.00	1.56
Tertiary	57 (4.87)	631.6 ± 14.9	140.36 ± 3.31	11.10	2.47	6.00	1.33	6.00	1.33
Nature of residence									
Rural	307 (26.2)	2,086.6 ± 8.7	463.69 ± 1.93	6.80	1.51	5.00	1.11	7.00	1.56
Urban	864 (73.8)	6,480.1 ± 16.9	1,440.02 ± 3.76	7.50	1.67	4.20	0.93	6.00	1.33
Paid for FP method									
Yes	1033 (88.29)	8,129.7 ± 15.7	1806.60 ± 3.49	7.90	1.76	5.00	1.11	7.40	1.64
No	137 (11.7)	437.00 ± 10.0	97.11 ± 2.22	3.20	0.71	0.50	0.11	4.00	0.89
Financing FP									
Self	668 (63.8)	4778.70 ± 8.3	1,061.93 ± 1.84	6.94	1.54	4.90	1.09	7.00	1.56
Spouse	362 (34.7)	3485.80 ± 23.9	774.62 ± 5.31	9.24	2.05	5.00	1.11	7.40	1.64
Other family member	16 (1.5)	302.30 ± 10.2	67.18 ± 2.27	10.09	2.24	6.00	1.33	11.50	2.56
Means of transport									
Walking	555 (47.4)	2512.9 ± 5.6	558.42 ± 1.24	4.50	1.00	2.50	0.56	4.00	0.89
Bicycle	162 (13.9)	891.6 ± 5.9	198.13 ± 5.59	5.50	1.22	4.00	0.89	6.50	1.44
Bus/taxi	417 (35.6)	4370.3 ± 12.3	971.18 ± 12.30	10.50	2.33	7.00	1.56	7.80	1.73
Private vehicle	36 (3.1)	791.9 ± 6.8	175.98 ± 6.8	23.00	5.11	2.75	0.61	8.75	1.94

*GHS* Ghanaian cedis, *USD* United States dollar, *IQR* Interquartile range, *SDP* Service delivery point, *FP* family planning

(–), No record of cost

Public Service Delivery Point: Government hospitals, clinics and health centers; Private Service Delivery Point: Private Hospitals, clinics and Health centers

#### Direct cost incurred for assessing family planning services

The total monetary cost for assessing FP services for the study population was estimated to be GHS 8,567.20 [US$ 1903.82], averaging at GHS 7.90 ± 5.7 [US$ 1.76 ± 1.27]. Fifty percent (median) of the clients incurred a cost of GHS 5.00 [US$ 1.11] per each visit to SDP. The total cost of contraceptive supplied was estimated at (GHS 4,588.30 [US$ 1,019.62]) translating to about 53.5% of the entire cost.

Fifty percent of clients spent GHS 3.00 [US$ 0.67] as client folder charges (That is monies paid before a client is issued a family planning folder for her first visit); GHS 10.00 [US$ 2.22] as consultation fee; GHS 5.00 [US$ 1.11] as Laboratory test/X-ray charges; GHS 2.00 [US$ 0.44] as contraceptive supplied and GHS 4.00 [US$ 0.89] as cost of transportation (Table 3).

**Table 3:** Summary of the direct cost for accessing family planning services from SDPs in Ghana

Cost segregation	Cost component					
	Client records Card (*n *= 114) [USD]	Consultation fee (*n *= 6) [USD]	Laboratory test/x-ray (*n *= 123) [USD]	Contraceptive supplied (*n *= 971) [USD]	Transportation cost (*n *= 505) [USD]	Total direct cost [USD]
Average (SD)	3.30 ± 2.2 [0.73 ± 0.49]	7.50 ± [1.67 ±]	6.03 ± 8.7 [1.34 ± 1.93]	4.73 ± 4.3 [1.05 ± 0.96]	5.50 ± [1.22 ±]	7.90 ± 5.7 [1.76 ± 1.27]
Median	3.00 [0.67]	10.00 [2.22]	5.00 [1.11]	2.00 [0.44]	4.00 [0.89]	5.00 [1.11]
IQR	3.00 [0.67]	6.00 [1.33]	1.00 [0.22]	4.00 [0.89]	3.00 [0.67]	6.5 [1.44]
Total (%)	368.50 [81.89]	45.00 [10.00]	742.00 [164.89]	4,588.30 [1,019.62]	2822.90 [627.31]	8,567.20 [1903.82]

*GHS* Ghanaian cedis, *USD* US Dollar rate at 2017 market price 1 US$ = *4.50*, *IQR* interquartile range

#### Indirect cost (e.g. time value lost)

Several productivity hours were lost in the course of accessing FP services. For instance, 46.8% stayed off paid work, 40.8% were not able to perform household chores, 11.5% lost relaxation time and 0.9% also had to forego other social engagement. The clients on average spent 54.21 min traveling at a distance of 3.49 km to SDPs and wait for 18.11 min before they are served (Additional file 1: Table S2).

### Discussion

The study aimed at estimating the hidden cost burden in accessing family planning services in Ghana. The findings contribute to the existing literature and discussed according to the direct and indirect costs.

#### Direct cost in accessing family planning

The cost of family planning services is relevant to clients, service providers and government policy implementers. Such a costing data could inform stakeholders to develop policies that aim to promote universal health financing, and subsequently promote universal health coverage. The study findings showed that clients spent an average direct cost of GHS 7.90 ± 5.7 [$1.76 ± 1.27] when accessing family planning services. The direct cost appears to be relatively high when compared with the standard of living and economic indicators. More specifically, the direct constitutes about 81% of the daily minimum wage of the Ghanaian working population (GHS 9.68 [$2.15]) in 2018 [43]. More specifically, the average expenditure on contraceptives and transportation to the facility was about 50% of the daily minimum wage. This suggests that a client spent relatively a daily income for FP services, a situation that could place a significant financial burden on the household economies of the ordinary Ghanaian. In most instance, users of modern contraceptives may feel reluctant and turn to traditional methods which have a high failure rate to delay pregnancies. This finding is consistent with previous studies in many LMICs countries, like Pakistan [44], India [17] and Bangladesh [19]. In these settings, the cost of health services (e.g. family planning) constitutes a major barrier in seeking care. In particular, the high cost of healthcare discourages clients with low-income status from seeking regular care and eventually opting for other cost-saving alternatives [6, 17, 18]. Given this challenge, National Health Insurance is increasingly introduced in many developing countries to remove out-of-pocket payments for clients. For instance, NHIS has been introduced into the Ghanaian health systems, yet the cost of FP is not covered under this policy. To achieve universal health financing, our study recommends that the current NHIS benefit should incorporate all family planning services. Future research should employ qualitative methods to explore the subjective effects of the cost burden on the pathways to FP.

Further, the study findings showed that there are differences in the cost burden of FP services according to the cost components. For instance, FP services at the tertiary level were higher above the average minimum wage compared to primary level facilities. Similarly, FP services were higher in private own facilities compared with public settings. Generally, the high cost of FP services at tertiary and private own facilities could be attributed to several reasons. Consistent with previous literature in LMICs, like India [19, 45], tertiary level and private own facilities are perceived to have adequate human resources and equipment to provide comprehensive services. Therefore, service providers at these settings intend to charge higher to improve the quality of FP services. The study recommends that government regulatory bodies should employ a universal fee across all levels of service provision for FP services. Future research should use interventional design to identify innovative ways of achieving equity in health financing for FP services.

#### Indirect cost of family planning

The study findings showed that consumers of the FP services lose a total of 73 min on each visit to SDP owing to traveling and waiting time. On each visit, the consumer travels an average distance of 3.5 km. Consistent with earlier studies [4, 5], FP services are unequally distributed across districts. Therefore, clients from rural communities may have to travel long distances to access FP services. The long traveling time to SDP could mean a loss in productive time for the clients. The study findings recommend that the government should employ innovative approaches such as telemedicine to improve access to FP services in rural districts. Also, future research should employ intervention studies to measure the effectiveness of telemedicine to promote FP services in rural districts.

## Limitations

The study was limited in terms of the data collection instrument. The data collection tool was self-developed and did not use any existing validated tool. Nevertheless, several scientific scrutinies such as pre-testing of tools, sampling facilities and respondents randomly, development of tools based on previous literature reviews of standard costing questionnaire, discussion of findings with relevant literature were employed.

## Supplementary information

**Additional file 1.** Additional tables.

## Data Availability

All dataset used for this study is in the custody of the corresponding author and will be made available upon reasonable request

## Abbreviations

FP: Family planning
GHS: Ghanaian cedis
NHIS: National Health Insurance scheme
ODK: Open data kit
RE: Resident enumerator
SDP: Service delivery point
US$: United States Dollar

## References

[1] Smith M, Keckley P (2017). Adding it up. Hosp Heal Netw.

[2] Ki-Moon B (2013). The millennium development goals report 2013.

[3] Alkema L, Kantorova V, Menozzi C, Biddlecom A (2013). National, regional, and global rates and trends in contraceptive prevalence and unmet need for family planning between 1990 and 2015: a systematic and comprehensive analysis. Lancet.

[4] Wirth ME, Balk D, Delamonica E, Storeygard A, Sacks E, Minujin A (2006). Setting the stage for equity-sensitive monitoring of the maternal and child health Millennium Development Goals. Bull World Health Organ.

[5] Asamoah BO, Agardh A, Östergren PO (2013). Inequality in fertility rate and modern contraceptive use among Ghanaian women from 1988–2008. Int J Equity Health.

[6] DeVoe JE, Baez A, Angier H, Krois L, Edlund C, Carney PA (2007). Insurance + access ≠ health care: typology of barriers to health care access for low-income families. Ann Fam Med.

[7] Adongo PB, Phillips JF, Binka FN (1998). The influence of traditional religion on fertility regulation among the Kassena-Nankana of Northern Ghana. Stud Fam Plann.

[8] Matlala SF, Mpolokeng MBL (2014). PNT: professional nursing today: official journal of the Forum for the profession of nurse leaders. Prof Nurs Today.

[9] Sedgh G, Hussain R (2014). Reasons for contraceptive nonuse among women having unmet need for contraception in developing countries. Stud Fam Plann.

[10] Williamson LM, Parkes A, Wight D, Petticrew M, Hart GJ (2009). Limits to modern contraceptive use among young women in developing countries: a systematic review of qualitative research. Reprod Health.

[11] Burke HM, Ambasa-Shisanya C (2011). Qualitative study of reasons for discontinuation of injectable contraceptives among users and salient reference groups in Kenya. Afr J Reprod Heal.

[12] Mugisha JF, Reynolds H (2008). Provider perspectives on barriers to family planning quality in Uganda: a qualitative study. J Fam Plan Reprod Heal Care.

[13] Singh S, Darroch JE (2013). Adding it up: the need for and cost of maternal and new-born care—estimates for 2012.

[14] Mills A, Borghi J, McIntyre D (2012). Equity in financing and use of health care in Ghana, South Africa, and Tanzania: implications for paths to universal coverage. Lancet.

[15] Harris B, Goudge J, Ataguba JE, McIntyre D, Nxumalo N, Jikwana S, Chersich M (2011). Inequities in access to health care in South Africa. J Public Health Policy.

[16] Asenso-Okyere WK, Anum A, Osei-Akoto I, Adukonu A (1998). Cost recovery in Ghana: are there Any changes in health care seeking behaviour?. Health Policy Plan.

[17] Griffiths P, Stephenson R (2001). Understanding users’ perspectives of barriers to maternal health care use in Maharashtra, India. J Biosoc Sci.

[18] Heller PS (1982). A model of the demand for medical and health services in Peninsular Malaysia. Soc Sci Med.

[19] Save the Children and Witter S. An Unnecessary Evil ? User fees for healthcare in An Unnecessary Evil ? User fees for healthcare in low-income countries. Save Child. Fund 2005 London. 2005.

[20] Witter S, Arhinful DK, Kusi A, Zakariah-Akoto S (2007). The experience of Ghana in implementing a user fee exemption policy to provide free delivery care. Reprod Health Matters.

[21] UNFPA/MOH (2004). Ghana health sector five year programme of work (2002–2006)-an in-depth review of the health sector response to maternal mortality in Ghana by 2003.

[22] Stephenson R, Hennink M (2004). Barriers to family planning service use among the urban poor in Pakistan. Asia-Pac Popul J.

[23] Machiyama K, Cleland J (2014). Unmet need for family planning in Ghana: the shifting contributions of lack of access and attitudinal resistance. Stud Fam Plann.

[24] Aviisah PA, Dery S, Atsu BK, Yawson A, Alotaibi RM, Rezk HR, Guure C (2018). Modern contraceptive use among women of reproductive age in Ghana: analysis of the 2003–2014 Ghana Demographic and Health Surveys. BMC Womens Health.

[25] Witter S, Adjei S, Armar-Klemesu M, Graham W (2009). Providing free maternal health care: ten lessons from an evaluation of the national delivery exemption policy in Ghana. Glob Health Action.

[26] Asante F, Chikwama C, Daniels A, Armar-klemesu M (2010). Evaluating the economic outcomes of the policy of fee exemption for maternal delivery care in Ghana. Ghana Med J.

[27] Badu E, Agyei-Baffour P, Ofori Acheampong I, Opoku MP, Addai-Donkor K (2019). Perceived satisfaction with health services under National Health Insurance Scheme: clients’ perspectives. Int J Health Plann Manage.

[28] Witter S, Armar-klemesu M, Dieng T (2008). National fee exemption schemes for deliveries: comparing the recent experiences of Ghana and Senegal.

[29] Ansong-tornui J, Armar-klemesu M, Arhinful D, Penfold S, Hussein J (2010). Hospital based maternity care in Ghana—findings of a confidential enquiry into maternal deaths. Ghana Med J.

[30] Ganle JK, Parker M, Fitzpatrick R, Otupiri E (2014). Inequities in accessibility to and utilisation of maternal health services in Ghana after user-fee exemption: a descriptive study. Int J Equity Health.

[31] Amissah J, Agyei-Baffour P, Badu E, Agyeman JK, Badu ED (2019). The cost of managing occupational injuries among frontline construction workers in Ghana. Value Heal Reg Issues.

[32] Kudebong M, Wurapa F, Nonvignon J, Norman I, Awoonor-Williams JK, Aikins M (2011). Economic burden of motorcycle accidents in Northern Ghana. Ghana Med J.

[33] Akazili J, Aikins M, Binka FN (2007). Malaria treatment in Northern Ghana: what is the treatment cost per case to households?. Afr J Health Sci.

[34] Sicuri E, Vieta A, Lindner L, Constenla D, Sauboin C (2013). The economic costs of malaria in children in three sub-Saharan countries: Ghana, Tanzania and Kenya. Malar J.

[35] Reforms HS. Overview of the Health System in Ghana. 1999.

[36] Aseweh Abor P, Abekah-Nkrumah G, Abor J (2008). An examination of hospital governance in Ghana. Leadersh Heal Serv.

[37] JeffreyCoker F, Basinger M. Open Data Kit: Implications for the Use of Smartphone Software Technology for Questionnaire Studies in International Development. Columbia Univ Mech Eng Dep Available online http//modi mech columbia edu/wp-content/uploads/2010/04/Open-Data-Kit-Review-Article pdf. 2010. Accessed 1 Jan 2015.

[38] Tom-Aba D, Olaleye A, Olayinka AT (2015). Innovative technological approach to ebola virus disease outbreak response in Nigeria using the open data kit and form hub technology. PLoS ONE.

[39] Tarricone R (2006). Cost-of-illness analysis: what room in health economics?. Health Policy (New York).

[40] Waehrer GM, Dong XS, Miller T, Haile E, Men Y (2007). Costs of occupational injuries in construction in the United States. Accid Anal Prev.

[41] Waehrer G, Leigh JP, Cassady D, Miller TR (2004). Costs of occupational injury and illness across states. J Occup Environ Med.

[42] Leigh JP, Miller TR (1998). Ranking occupations based upon the costs of job-related injuries and diseases. Occup Heal Ind Med.

[43] Nyavor G. Minimum wage goes up by 10%; now GHS10.65. In: Myjoyonline.com. 2018. https://www.myjoyonline.com/business/2018/July-27th/minimum-wage-goes-up-by-now-gh1065.php. Accessed 27 Aug 2019.

[44] Stephenson R, Hennink M Barriers to Family Planning Service use among the Urban Poor in Pakistan.

[45] Chatterjee S, Levin C, Laxminarayan R (2013). Unit cost of medical services at different hospitals in India. PLoS ONE.

