# Supplementary material for: In search of universal health coverage: the hidden cost of family planning to women in Ghana
Source: BMC Res Notes. 2020 Feb 7;13:58. doi: 10.1186/s13104-020-4928-2 (PMC7006161; doi:10.1186/s13104-020-4928-2)
Supplement: Supplementary file 1 — Additional file 1. Additional tables. [file 13104_2020_4928_MOESM1_ESM.docx]

**Additional Table S1: Proportion of sample population**

| **Type of facility** | **Total no of institutions** | **Proportion** | SDPs Sampled |
| --- | --- | --- | --- |
| Primary Facilities | 3,107 | 0.936407 | 210 |
| Secondary Facilities | 207 | 0.062387 | 121 |
| Tertiary Facilities | 4 | 0.001206 | 5 |
| Totals | 3,318 | 1.000000 | 336 |

**Additional Table S2: Time value lost by clients owning to accessing family planning services**

| **Variable** | **Frequency** | **Unit** |
| --- | --- | --- |
| **Distance traveled from residence to SPD** | *n=1194* | kilometers |
| Average |  | 3.49 |
| Median |  | 2.0 |
| IQR |  | 1.2 |
| Total |  | 4,081.56 |
| **Waiting time at SDP** | *n*=1190 | minutes |
| Average |  | 18.11 |
| Median |  | 10.00 |
| IQR |  | 15.00 |
| Total |  | 21,186.45 |
| **Total travel time to SDP** | |  |
| Average |  | 54.21 |
| Median |  | 40 |
| IQR |  | 40 |
| Total |  | 63,430.25 |
| **Productive activity forgone** | *n=1170* | **Percentage** |
| Household chores | 477 | 40.8 |
| Paid work | 547 | 46.8 |
| Resting | 135 | 11.5 |
| Other social engagement | 11 | 0.9 |
| **IQR**: Interquartile range  **SDP**: Service delivery point |  |  |
|  |  |  |
